# Aggressions in psychiatric settings: Profile of victims and circumstances

**DOI:** 10.1192/j.eurpsy.2026.11520

**Published:** 2026-07-31

**Authors:** A. Hrairi, F. Dhouib, N. Kotti, N. Remadi, M. Hajjaji, K. Jmal Hammami

**Affiliations:** Occupational medecine Department, Faculty of medecine of Sfax, Sfax, Tunisia

## Abstract

**Introduction:**

According to the World Health Organization, more than half of healthcare workers are likely to have experienced acts of violence. Healthcare workers in psychiatric settings are considered to be at risk.

**Objectives:**

We had conducted a study aiming to determine the profile of victims of aggression in psychiatric settings and to study the circumstances surrounding these aggressions.

**Methods:**

It was a descriptive retrospective study conducted among paramedical staff working in the psychiatric wards of Hedi Chaker University Hospital, who had been victims of aggression reported as occupational injuries.

**Results:**

Our population consisted of 31 psychiatric staff members with an average age of 40.7 years. The majority of these victims were nurses (64.5%). The average tenure in psychiatric care was 13.6 years. Most of these aggressions (61.3%) occurred after the Tunisian revolution. The aggressor was the patient in 97% of cases. Acts of violence mainly occurred in the morning (45%). The nature of the injuries was especially bruises (58%) and wounds (16.1%). These injuries were located mainly in the upper limb in 41.9% of cases and in the trunk in 35.4% of cases.

**Conclusions:**

Given the characteristics of patients and the care provided, aggression in psychiatric settings can have serious consequences on the physical and mental well-being of healthcare worker. The implementation of appropriate prevention measures, as well as training and information for staff, is necessary for better management of violence in this specific environment.

**Disclosure of Interest:**

None Declared

